# Comparative transcriptomic analysis reveals an association of gibel carp fatty liver with ferroptosis pathway

**DOI:** 10.1186/s12864-021-07621-2

**Published:** 2021-05-05

**Authors:** Xiao-Juan Zhang, Li Zhou, Wei-Jia Lu, Wen-Xuan Du, Xiang-Yuan Mi, Zhi Li, Xi-Yin Li, Zhong-Wei Wang, Yang Wang, Ming Duan, Jian-Fang Gui

**Affiliations:** 1 College of Fisheries, Huazhong Agricultural University, Wuhan, 430070 China; 2 State Key Laboratory of Freshwater Ecology and Biotechnology, Institute of Hydrobiology, Innovation Academy for Seed Design, Chinese Academy of Sciences, Wuhan, 430072 Hubei China; 3 University of Chinese Academy of Sciences, Beijing, 100049 China

**Keywords:** Fatty liver, Comparative transcriptome, Latent pathway, Ferroptosis, Gibel carp

## Abstract

**Background:**

Fatty liver has become a main problem that causes huge economic losses in many aquaculture modes. It is a common physiological or pathological phenomenon in aquaculture, but the causes and occurring mechanism are remaining enigmatic.

**Methods:**

Each three liver samples from the control group of allogynogenetic gibel carp with normal liver and the overfeeding group with fatty liver were collected randomly for the detailed comparison of histological structure, lipid accumulation, transcriptomic profile, latent pathway identification analysis (LPIA), marker gene expression, and hepatocyte mitochondria analyses.

**Results:**

Compared to normal liver, larger hepatocytes and more lipid accumulation were observed in fatty liver. Transcriptomic analysis between fatty liver and normal liver showed a totally different transcriptional trajectory. GO terms and KEGG pathways analyses revealed several enriched pathways in fatty liver, such as lipid biosynthesis, degradation accumulation, peroxidation, or metabolism and redox balance activities. LPIA identified an activated ferroptosis pathway in the fatty liver. qPCR analysis confirmed that *gpx4*, a negative regulator of ferroptosis, was significantly downregulated while the other three positively regulated marker genes, such as *acsl4*, *tfr1* and *gcl*, were upregulated in fatty liver. Moreover, the hepatocytes of fatty liver had more condensed mitochondria and some of their outer membranes were almost ruptured.

**Conclusions:**

We reveal an association between ferroptosis and fish fatty liver for the first time, suggesting that ferroptosis might be activated in liver fatty. Therefore, the current study provides a clue for future studies on fish fatty liver problems.

**Supplementary Information:**

The online version contains supplementary material available at 10.1186/s12864-021-07621-2.

## Background

Fish fatty liver is a common physiological or pathological phenomenon in aquaculture. The causes are complex and not well-known, and mainly include imbalance nutrition diet, environmental pollutants, physiological factors and genetic mutation [1]. Fatty liver diseases have been found in most main farmed fish, and caused many problems, such as low feed efficiency, immune response, flesh and nutritional quality effects [1–5]. The utilization of artificially formulated feeds can bring nutritional, physiological and ecological effects to fish [2, 6–9]. However, exceeding nutrition, improper artificial formulated diets and overfeeding have led to a growing concern of liver fatty problems, such as hepatocyte enlargement, lipid accumulation, steatosis, fibrosis and necrosis [10, 11].

Fatty liver can be mainly classified as “nutritional fatty liver” or “oxidative fatty liver” in aquaculture. Nutritional fatty liver, principally caused by imbalanced nutrition supply, is common in farmed fish and generally not a pathological symptom. It can be alleviated through adjusting diet formulation or feeding. If no effective strategy was carried on, it could be turned to the “oxidative fatty liver” or directly induced to hepatic fibrosis and necrosis, which causes irretrievable damages [1]. Oxidative stress might lead to oxidative fatty liver, which would arouse severe liver damage [1, 12]. Overall, excess dietary energy intake and severe peroxidation can cause fish metabolic imbalance and then result in fatty livers. Some researchers suggest that many pathways, such as target-of-rapamycin complex 1 (Torc1), AMP-activated protein kinase (AMPK), transcription factor EB (TFEB), peroxisome proliferator activated receptor (PPAR), P53, nuclear erythroid 2-related factor 2 (Nrf2), c-jun Nterminal kinase (JNK), toll-like receptors (TLRs), myeloid differentiation primary-response protein 88 (Myd88), and nuclear factor κB (NF-κB) signaling pathways, might be related to fatty liver caused by high-fat/carbohydrate or over-nutrition diet [11–17]. For example, the decreased AMPK pathway can suppress autophagy and then worsen lipotoxicity in tilapia fatty liver [11], and its activation can reduce the expression of genes related to lipogenesis in rainbow trout liver [16]. However, some results seem to be controversial. For instance, the activation of Nrf2, JNK and TLRs-Myd88-NF-κB signaling pathways could lead to inflammation and worsen tilapia liver injury [12], while cAMP-JNK/NF-κB-caspase signaling pathway could protect the fatty liver tissues from more serious damage though regulating the hemostasis phosphorylation of JNK protein in Japanese seabass [17]. JNK and NF-κB signaling pathways might play a dual role in the fish fatty liver. Therefore, the mechanism of hepatic lipid accumulation and fish fatty liver are remaining enigmatic. In addition, too many affected pathways were identified and few studies had explored which key pathway was associated with fish fatty live.

Gibel carp (*Carassius gibelio*) is one of the most important aquaculture species in China [18–23] and the production yields in China have increased to 2,755,632 tons in 2019 [24]. In lotus-fish farming ponds, we found some individuals of gibel carp had fatty liver. To find out the cause, we first analyzed the liver histological structures and lipid accumulation of normal liver and fatty liver. Then, we conducted comparative transcriptomic analysis between, and identified a pathway of ferroptosis that was significantly activated in fatty liver. Finally, we confirmed that ferroptosis was activated in fatty liver by qPCR analysis and mitochondria morphological observation. Our current study establishes an association between ferroptosis and fish liver fatty, which provides a clue for future studies on fish liver fatty problems.

## Results

### Morphological changes in fatty liver

**Fig. 1:**
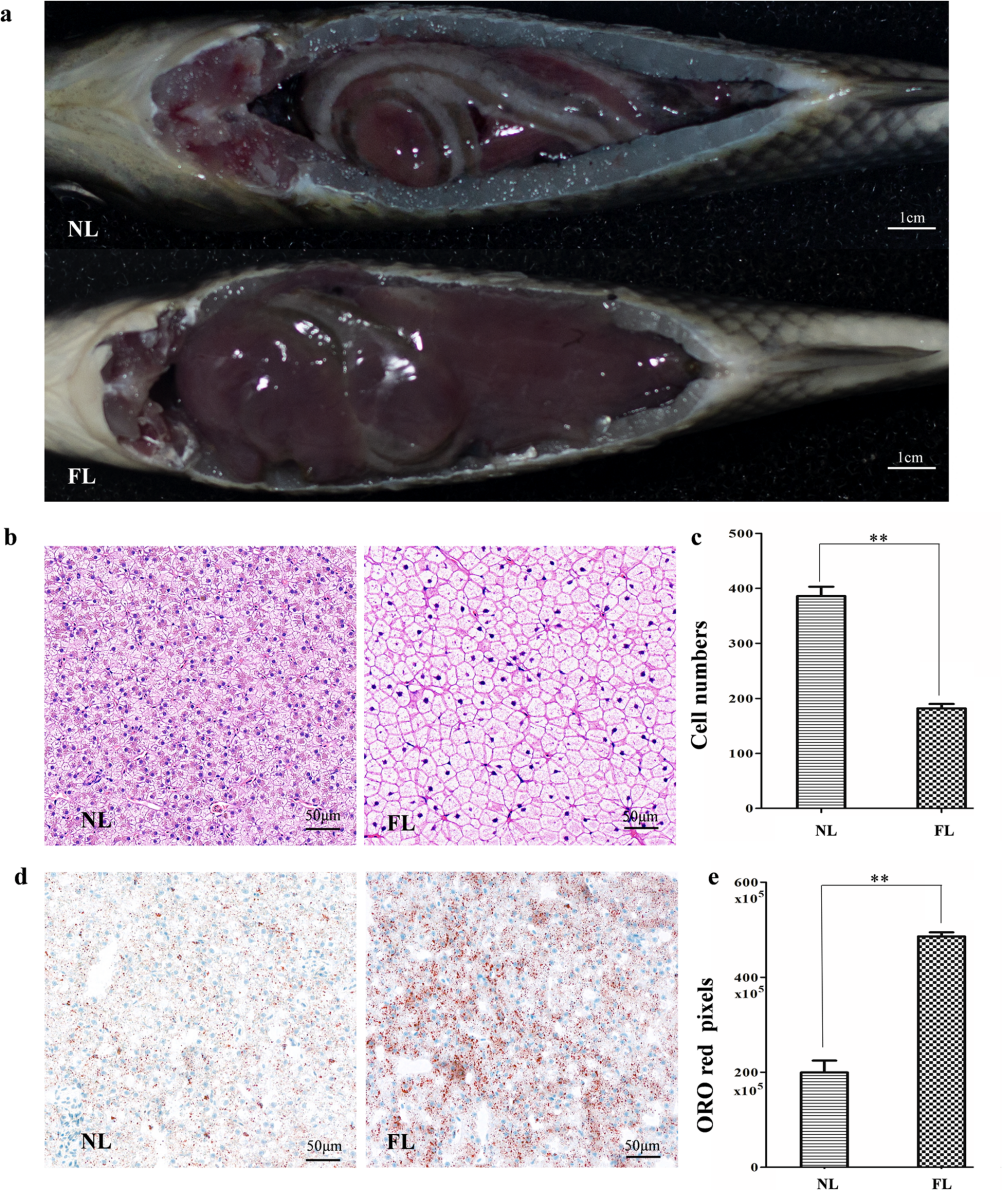
Histology and lipid accumulation of gibel carp normal liver and fatty liver. **a** Liver morphology of gibel carp. **b** Historical structure of liver tissues. The black column is scale bar (50 μm). **c** Number of hepatocytes on an area of 10 μm square. **d** ORO staining of liver tissues. Red color indicates lipid droplets. The black column is scale bar (50 μm). **e** ORO red pixels (X 10^5^) in equivalent area. Asterisks stand for the significant differences between normal liver and fatty liver (**: *p* < 0.01). NL: normal liver, FL: fatty liver

The morphology of fatty liver showed obviously different from that of normal liver (Fig. 1a). The whole liver was more hypertrophic and the hepatocytes of fatty liver were more enlarged (Fig. 1b), showing less hepatocytes on an area of 10 μm square (Fig. 1c) (*p* < 0.01). And then, we performed oil red O staining (ORO) to compare the lipid accumulation between normal liver and fatty liver. As we expected, fatty liver showed more than 2 times lipid accumulation than that in normal liver (Fig. 1d-e) (*p* < 0.01).

### Transcriptomic differences between normal liver and fatty liver

The transcriptomes of six liver samples in two groups were obtained using BGISEQ-500 Iillumina sequencing plat and each sample produced an average of 10.16 Gb clean bases. An average of 83.42 and 69.46% clean reads were mapping to the gibel carp’ genome and gene sets (Table 1).

**Table 1:** Summary statistics of sequencing data

Sample	Total Raw Reads (M)	Total Clean Bases (Gb)	Clean Reads Q20 (%)	Clean Reads Q30 (%)	Clean Reads Ratio (%)	Total Mapping (%)	Uniquely Mapping (%)
NL-1	77.13	10.07	96.50	88.09	87.05	83.05	47.00
NL-2	77.13	10.14	96.50	88.18	87.61	81.42	45.64
NL-3	77.13	10.22	96.60	88.4	88.37	81.56	45.11
FL-1	73.62	10.27	96.55	88.09	93.01	85.16	46.19
FL-2	73.62	10.10	96.58	88.16	91.43	84.83	47.37
FL-3	73.62	10.19	96.68	88.44	92.26	84.47	45.95

Note: *NL* normal liver, *FL* fatty liver

Finally, a total of 37,077 unigenes were obtained, which included 32,679 known genes and 4398 novel genes. Principal component analysis (PCA) showed groupings between normal liver and fatty (Fig. S1a). The correlation coefficient (R^2^) is > 0.93 in group and < 0.83 between groups (Fig. S1b), suggesting the best-fitting regression line for the technical replicates according to standards and best practices for RNA-Seq. The results demonstrate that though there are individual differences, the two groups have a totally different transcriptional trajectory (Fig. S1) [25, 26].

### Enriched GO terms in fatty liver

**Fig. 2:**
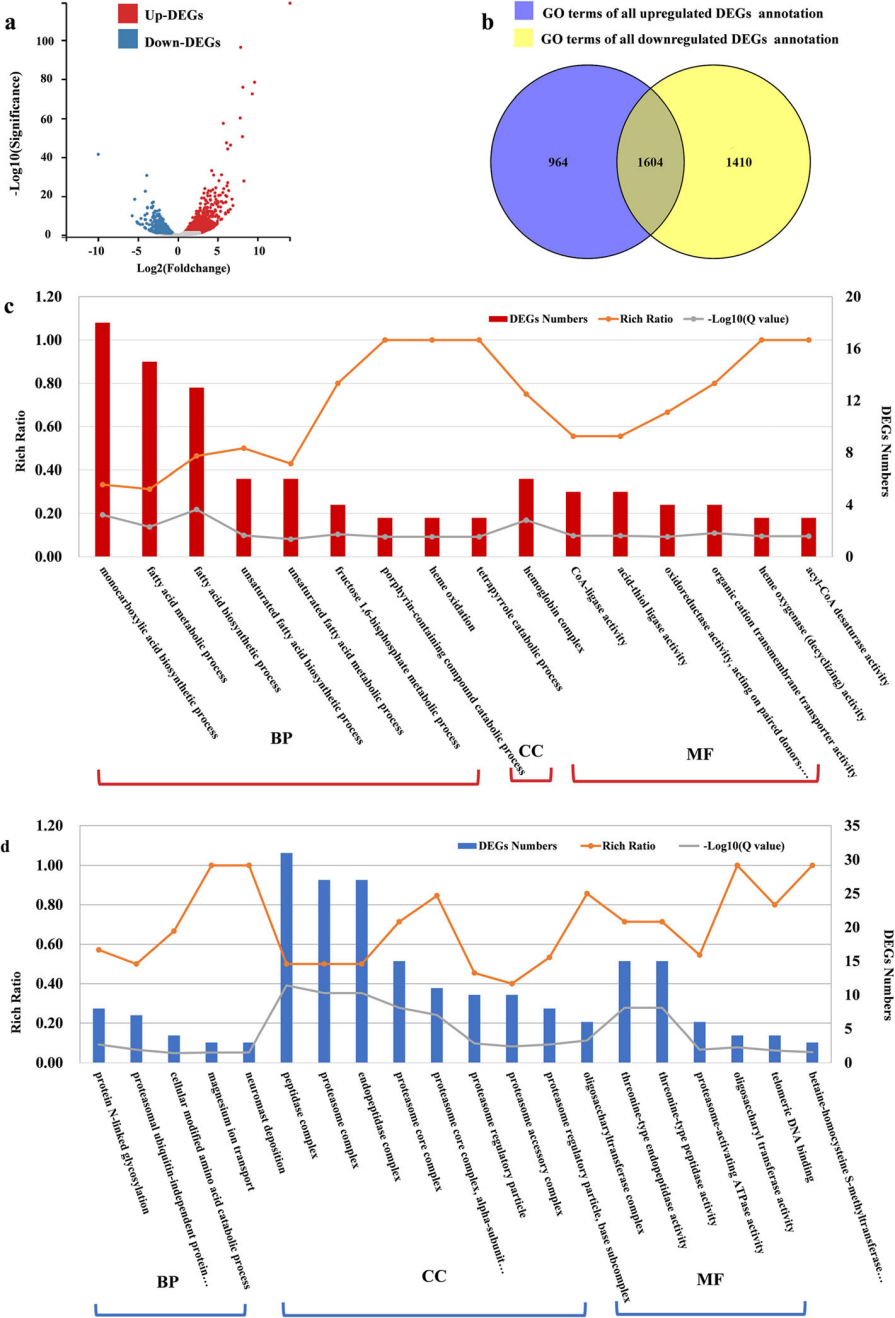
DEGs and GO enrichment analysis of gibel carp normal liver and fatty liver. **a** Gene expression patterns between two groups. **b** Venn’s diagrams visualize the up or downregulated DEGs associated with GO terms. **c** 16 enriched GO terms with all up- regulated DEGs (q-value≤0.05). **d** 20 enriched GO terms with all down- regulated DEGs (q-value≤0.05). BP: biological processes. CC: cellular component. MF: molecular functions. NL: normal liver, FL: fatty liver

A total of 3480 differentially expressed genes (DEGs) were assigned with the correction of *p*-values using FDR ≤ 0.001 (Table S1). Compared to normal, 1997 genes in fatty liver were upregulated and 1483 genes were downregulated (Fig. 2a). All up or downregulated DEGs were separately annotated into 2568 GO Gene Ontology (GO) terms and 3014 GO terms, among them, as visualized in Venn’s diagrams, 964 and 1410 GO terms were just associated with up or downregulated DEGs (Fig. 2b). Sixteen and 20 GO terms were significantly enriched with the correction of q-value ≤0.05 (Fig. 2 c-d).

Sixteen significantly enriched GO terms with all upregulated DEGs (Table S2), were involved in nine “biological processes” (BP), one “cellular component” (CC), six “molecular functions” (MF) (Fig. 2c). Four GO terms, “fatty acid metabolic process” (15 DEGs, GO 0006631), “fatty acid biosynthetic process” (13DEGs, GO 0006633), “unsaturated fatty acid biosynthetic process” (6 DEGs, GO 0006636), “unsaturated fatty acid metabolic process” (6 DEGs, GO 0033559), gave a direct hint that lipid synthesis or metabolism were active. Other upregulated GO terms were assigned to “fructose”, “heme”, “oxidoreductase activity”, “hemoglobin complex”, and “CoA-related activity”. And these genes might play important roles in the lipid accumulation, fatty acid transport and oxidation, redox balance, or other metabolic regulatory processes [13, 27–29].

Twenty significantly enriched GO terms with all downregulated DEGs (Table S2), were involved in 5 BP, 9 CC, and six MF (Fig. 2d). Most of the downregulated GO terms were related to “proteasome” or “peptidase”, such as “proteasome complex” (27 DEGs, GO 0000502), “proteasome core complex” (15 DEGs, GO 0005839), “proteasome accessory complex” (10 DEGs, GO 0022624), “proteasome-activating ATPase activity” (6 DEGs, GO 0036402), “peptidase complex” (31 DEGs, GO 1905368), “endopeptidase complex” (27 DEGs, GO 1905369), and “threonine-type peptidase activity” (15 DEGs, GO 0070003). Enzymes of them could catalyze biological reactions rapidly and unidirectionally regulate diverse basic cellular activities [30], suggesting some catalytic reactions might slow down in fatty liver.

### Significant pathways revealed by KEGG and LPIA in fatty liver

**Fig. 3:**
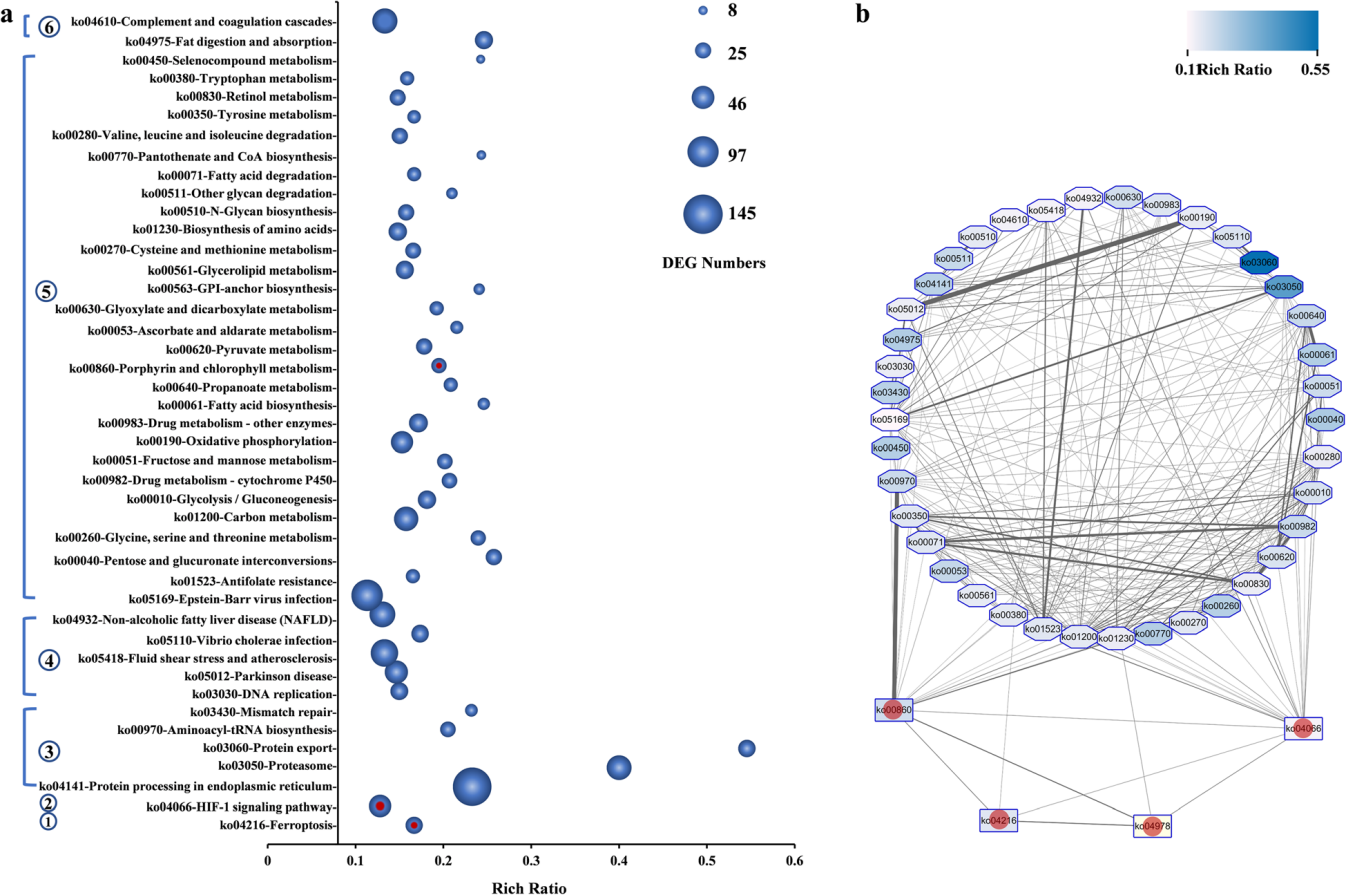
KEGG pathway enrichment analysis and pathway-pathway network. **a** All enriched KEGG pathways (q-values≤0.05). The x-axis indicates the rich factor of each pathway and y-axis shows pathway. The size of ball indicates the numbers of DEGs assigned to the corresponding pathway respectively. ①: cellular process category. ②:environmental information processing category. ③: genetic information processing category. ④: human diseases (just animal) category. ⑤: metabolism category and ⑥: organismal systems category. **b** Enriched KEGG pathways and latent pathways network (weight scores> 0.1). Latent pathways are marked with red dot. Line width stands for the weight between two pathways. Colors stands for rich ratio in KEGG pathways enrichment method

A total of 2194 DEGs were assigned to 336 Kyoto Encyclopedia of Genes and Genomes (KEGG) pathways [31], and some (43) of them were significantly enriched with the correction of q-value ≤0.05 (Fig. 3a; Table S3) and functionally divided into six categories, including one “cellular process”, one “environmental information processing”, six “genetic information processing”, six “diseases”, 27 “metabolisms”, and two “organismal systems”. Five enriched pathways, such as “Ferroptosis” (29 DEGs, ko04216), “Non-alcoholic fatty liver disease (NAFLD)” (65DEGs, ko04932), “Fatty acid biosynthesis” (15DEGs, ko00061), “Fatty acid degradation” (20DEGs, ko00071), “Glycerolipid metabolism” (32DEGs, ko00561), and “Fat digestion and absorption” (32DEGs, ko04975), were directly associated with lipid biosynthesis, degradation, peroxidation, or metabolism [32, 33].

**Fig. 4:**
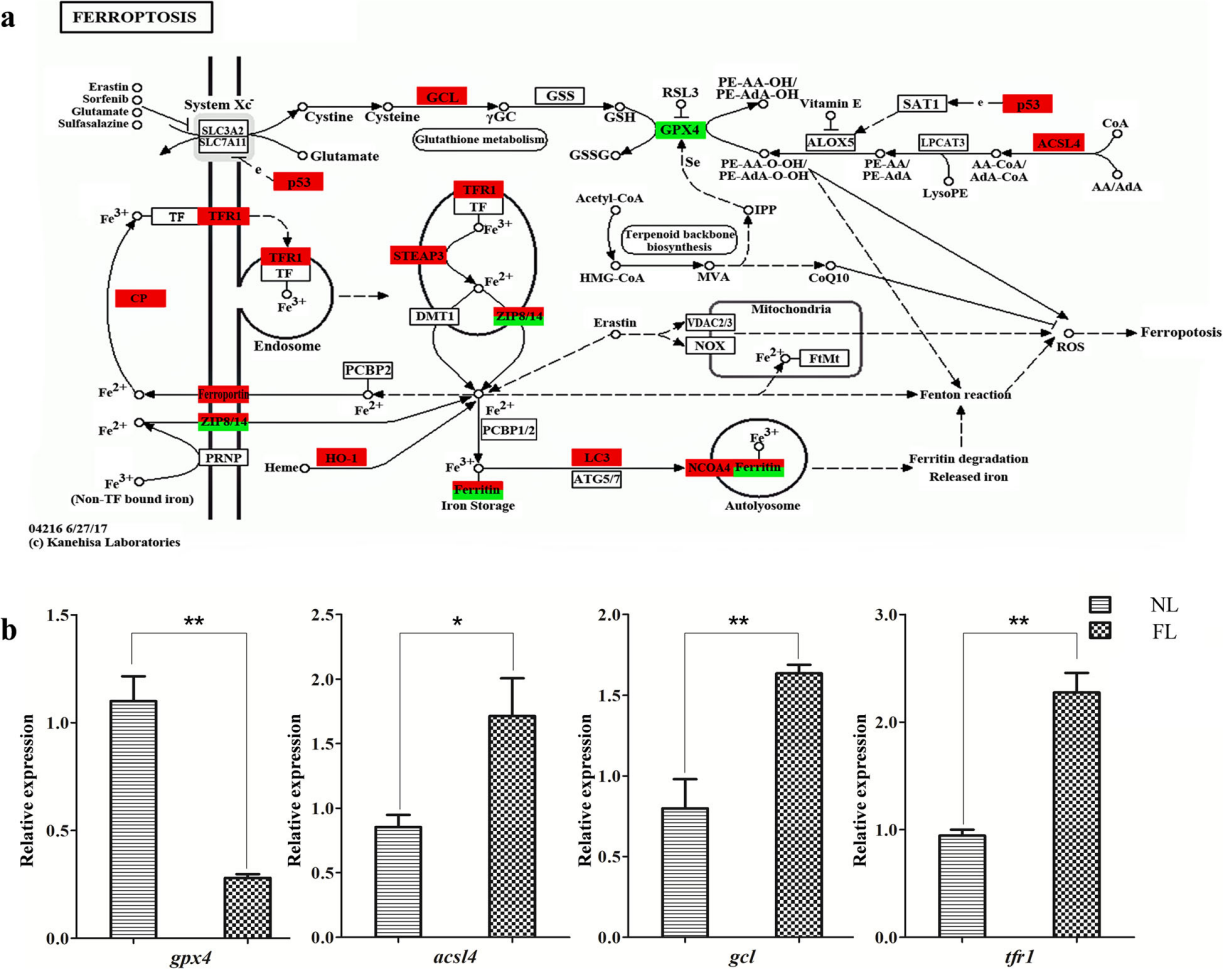
Ferroptosis in fatty liver and verification of biomarker genes by qPCR. **a** DEGs in ferroptosis in comparative transcriptomic analysis (29 DEGs, ko04216, https://www.genome.jp/dbget-bin/www_bget?map04216). Up and downregulated DEGs are shown in red and green, respectively. **b** The relative expression of *gpx4*, *acsl4*, *tfr1* and *gcl* in qPCR analysis. *β-actin* is used as the normalizer. Each bar represents mean ± standard deviation (SD) (*n* = 3). Asterisks stand for the significant differences between normal liver and fatty liver. (*: *p* < 0.05 and **: *p* < 0.01). NL: normal liver, FL: fatty liver

According to the LPIA method, a total of 316 KEGG pathways and 962 GO terms with biological process classification, which two shared at least 1 DEG, were selected to conduct the pathway-pathway interaction network (Table S4). Four significant pathways were identified with LPIA_v_1.pl [34] in Perl with 1000 iterations (Table 2), such as “Porphyrin and chlorophyll metabolism” (23 DEGs, ko00860) (Fig. S2a), “Hypoxia-inducible factor-1(HIF-1) signaling pathway” (49 DEGs, ko04066) (Fig. S2b), “Ferroptosis” (29 DEGs, ko04216) (Fig. 4a), and “Mineral absorption” (17 DEGs, ko04978) (Fig. S2c).

**Table 2:** The significant pathways in pathway-pathway interaction network by LPIA method

Pathways	Adjusted ***p***-value^**1**^	Rich Ratio^**2**^	q-value^**2**^
ko00860-Porphyrin and chlorophyll metabolism	0.00	0.1949	0.0028
ko04066-HIF-1 signaling pathway	0.00	0.0296	0.0033
ko04216-Ferroptosis	0.00	0.1667	0.0057
ko04978-Mineral absorption	0.00	0.1104	0.5570*

Note: Adjusted *p*-value^1^ was calculated in LPIA method;

Rich Ratio^2^ and q-value^2^ were calculated in KEGG pathways enrichment method

After the weight Aij was calculated at random walk, a correlational network was conducted between pathways that included 43 enriched pathways in KEGG enrichment analysis and four significant latent pathways (marked with red point) in LPIA method (Table S5). It was clearly showed that four significant latent pathways connected with each other in the network (weight scores > 0.1) (Fig. 3b). The pathways “Porphyrin and chlorophyll metabolism” (23 DEGs, ko00860) (Fig. S2a) and “HIF-1 signaling pathway” (49 DEGs, ko04066) (Fig. S2b) had more connections with other pathways in KEGG enrichment analysis. Among them, excepting *ZIP8/14* & *Ferritin* with a up & down expression, *glutathione peroxidase 4* (*gpx4*), a negative regulated gene in “Ferroptosis” [35–37], was downregulated, while other DEGs in “Ferroptosis” were all upregulated (Fig. 4a). The results suggest that the expression levels of genes in pathway “Ferroptosis” might be significantly different between normal liver and fatty liver.

### Ferroptosis is activated in fatty liver

To validate the different expressions of genes in “Ferroptosis” between normal liver and fatty liver, four marker genes, *gpx4* [35–40], *acyl-CoA synthetase long-chain family member 4* (*acsl4*) [41, 42], *transferrin receptor 1* (*tfr1*) [43], and *Glutamate-cysteine ligase* (*gcl*) [44], were selected for qPCR analysis. Consistent with the transcriptome result, *gpx4* was downregulated, while the others were all upregulated (Fig. 4b).

**Fig. 5:**
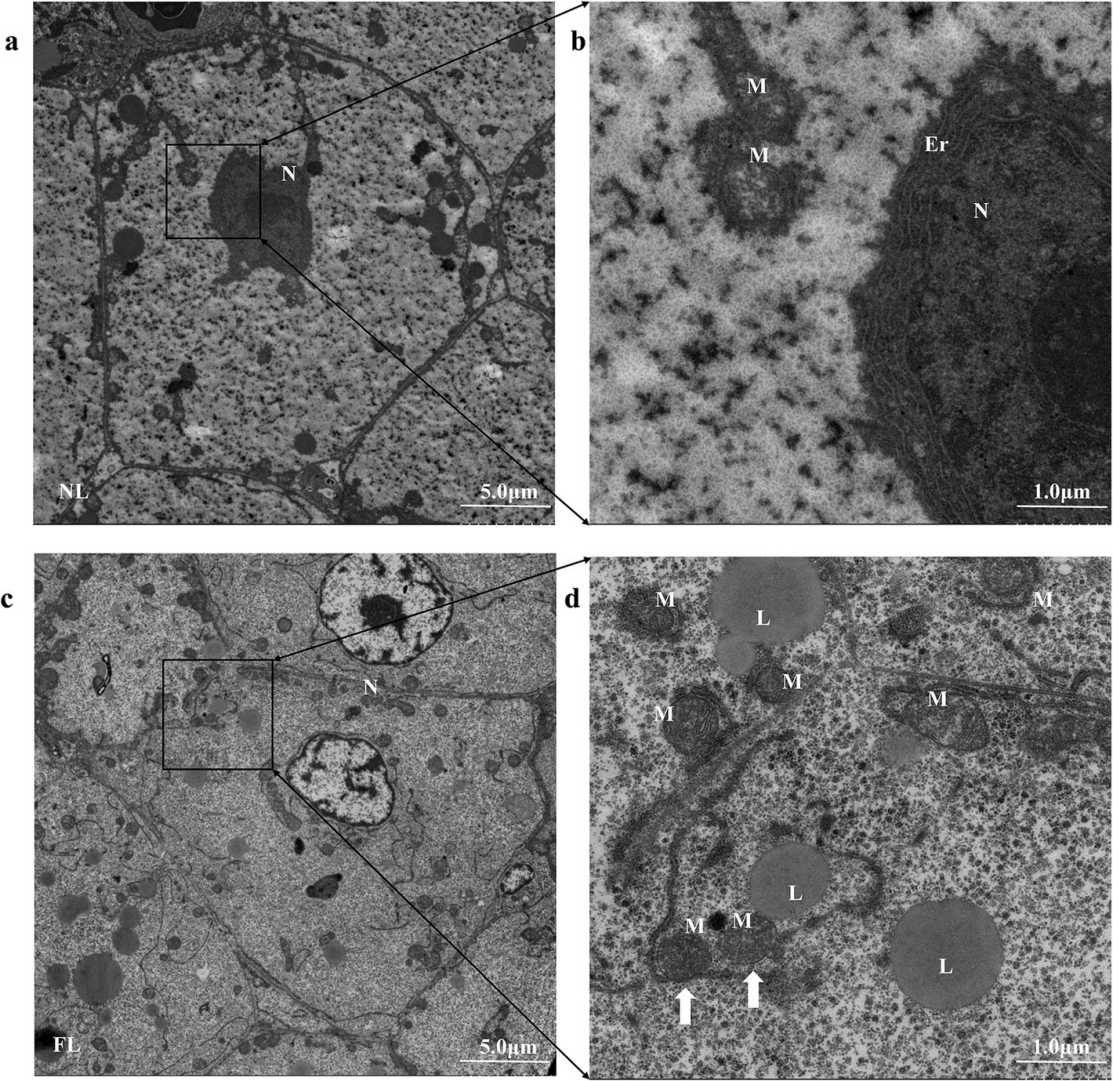
Electron micrographs of hepatocytes of gibel carp normal liver and fatty liver. **a-b** Electron micrographs of hepatocytes in normal liver; **c-d** Electron micrographs of hepatocytes in fatty liver. Er: endoplasmic reticulum. L: lipid droplet. M: mitochondria. N: nucleus. The white arrows indicate the condensed mitochondria with outer membrane ruptured. The white scale bar stands for 5 μm in a & c, and 1 μm in b &d. NL: normal liver, FL: fatty liver

Ferroptosis can also be characterized with the morphology of mitochondria [32, 45]. To observe the morphological differences of mitochondria between fatty liver and normal liver, we performed transmission electron microscopy analysis. There was no significant cellular dysfunction, but compared to normal liver, the mitochondria densities in fatty liver were more condensed, and some of outer mitochondrial membrane had been ruptured and seemed like single-membrane (Fig. 5). Taken together, ferroptosis was more sensitive in the fatty liver.

## Discussion

In this study, we found morphological changes in fatty liver, such as hepatocyte enlargement and lipid accumulation (Fig. 1). GO and KEGG enrichment analysis showed that activities related to lipid biosynthesis, degradation, accumulation, peroxidation or metabolism were more active in fatty liver (Fig. 2e & Fig. 3a). Importantly, a pathway of ferroptosis was significant different between normal liver and fatty liver that might be associated with lipid-related activities (Figs. 4 & 5), suggesting an association between ferroptosis and fish fatty liver.

Based on the current data, we suggest that a significant pathway of ferroptosis might be associated with fish fatty liver. Ferroptosis is actually an iron-catalyzed-lipid peroxidation disorder, and is related with lipid peroxidation. Previous research in fish suggested a possible link between ferroptosis and lipid. For example, the iron-catalyzed lipid peroxidation was characterized in zebrafish and cultured shrimp [46, 47]. Enzymes, such as glutathione peroxidase, could reduce the lipid peroxides in tilapia and other fish [48, 49]. Oxidants could damage mitochondrial membrane permeability and electron transport chain integrity in zebrafish and grass carp [50–52]. However, ferroptosis in fish is not clear. Now, we establish an association between ferroptosis and fatty liver through comparative transcriptomic analysis, marker gene expression and mitochondrion morphology observation in fish.

Ferroptosis is a new form of regulated cell death that depends on iron- and lipid-based reactive oxygen species (ROS), and has been implicated in both normal and pathological physiology which involves in various biological contexts of diverse species increasingly and widely [32, 35, 38, 53]. It is totally different from other reported forms of cell death, such as apoptosis, autophagy, necrosis and pyroptosis [40, 53, 54], and associated with various liver problems [38, 55]. In fish, it potentially was responsible for the lethality of zebrafish VitE-deficient embryos [56]. It might be activated by low temperature (Nile tilapia), hypoxia (Silver sillago) stress and heavy metal (Japanese flounder) [57–59], and could be induced by *Escherichia coli* and then led to the red blood cells (RBCs) death of grass carp [60]. It could be checked at the gene expression level and characterized by the morphology of mitochondria [32, 35, 36, 38, 39, 41–45, 61]. Some genes in ferroptosis are classified as markers. For example, *gpx4* is required for the clearance of lipid ROS. If it was inactivated or inhibited, lipid ROS would accumulate, and then induced ferroptosis [35–40]. *acsl4*, as an essential component for ferroptosis execution, could dictate ferroptosis sensitivity. The more upregulated expression of *acsl4*, the more sensitive to ferroptosis [41, 42]. In this study, compared to normal liver, the relative expression of negative regulator *gpx4* was significantly downregulated and the relative expression of three positive actors (*acsl4*, *tfr1* and *gcl*) were significantly upregulated, implying that fatty liver was more sensitive to ferroptosis. In addition, ferroptosis showed smaller, condensed and outer membrane ruptured of mitochondria with crista diminished or vanished in cellular morphological characteristics [62, 63]. Moreover, the GO and KEGG enrichment analysis suggest lipid-related activities were active in fatty liver. Therefore, our results indicate that ferroptosis might be associate with liver fatty and it is different between normal liver and fatty liver.

The main method used in this study was LPIA, which was more appropriate to our study in one challenge with metabolic relevant. It could give a direct understanding to the key cellular mechanisms in biological activities [34, 64]. In this study, four latent pathways were identified by LPIA (Table 2), including ferroptosis. Among them, the remaining three latent pathways might also connect with ferroptosis (Fig. 3b). For example, “Porphyrin and chlorophyll metabolism” pathway was associated with the energy, ion and erythroid heme synthesis regulation, especially in responding to various stresses [65, 66]. “HIF-1 signaling” pathway was involved in cellular responses to low oxygen environments and growth factors [67, 68]. It was noteworthy that both “Porphyrin and chlorophyll metabolism” and “HIF-1 signaling” pathways had more relations with other enriched pathways (Fig. 3b). Minerals were fundamental nutrients and mineral absorption may depend on dietary ingredient composition. Some genes in “mineral absorption pathway” were involved in iron metabolism [69, 70]. These could explain why they were all identified and suggest that ferroptosis may be the key pathway involved in fatty liver.

Overall, as lipid accumulation continued to increase, lipid peroxidation would also increase [52]. If hepatocytes could not be sufficient capacity to eliminate lipid peroxides, ferroptosis might be activated [32, 53]. Certainly, more research on the mechanism underlying ferroptosis and fish fatty liver need to be carried out. Since ferroptosis could be regulated and numbers of small molecule inhibitors, such as Vitamin E, the nature’s most-efficient ferroptosis inhibitor, have been identified [32, 33, 40, 56, 71–73], which suggests new prevention strategies and promising therapies of fish fatty liver.

## Conclusion

Based on detailed comparison of histological structure, lipid accumulation, transcriptomic profile, ferroptosis marker gene expression, and hepatocyte mitochondria between normal liver and fatty liver, our study reveals an association between ferroptosis and fatty liver in fish for the first time, suggesting that ferroptosis might be activated in fish liver fatty. The current study provides a clue for future studies on fish fatty liver and effective prevention strategies in fish fatty liver problems.

## Materials and methods

### Animals and sample collection

All experimental procedures in this study were performed in accordance with the guidelines and after approval of the Animal Care and Use Committee of Institute of Hydrobiology, Chinese Academy of Sciences (IHB, CAS, Protocol No. 2016–018). A total of 3000 juveniles of allogynogenetic gibel carp, which came from same clones, were transported from National Aquatic Biological Resource Center of Institute of IHB, CAS, which is located in Wuhan, China, to the Luo-Fu Lotus Farm, which is located in Jinggangshan, China, for lotus-fish culturing. Fifty juveniles were selected randomly to weight (Table S6). Half of the juveniles was set as an overfeeding group, which was continuing feeding until without fish activities in apparent satiation two times per day. The other half was set as a control group, which was cultured without artificial feeding. All the juveniles were cultured in lotus-fish culturing ponds at a density of 150 juveniles per 667 m^2^. Water samples were collected from three randomly ponds three times (April 22th, June 3th, October 12th, 2019). Eight physicochemical water quality parameters (water temperature, dissolved oxygen, pH, total nitrogen, ammonia, nitrate, nitrite and total phosphorus) were analyzed (Table S7). After 180 days, commercial fish were harvested. Fifty commercial fish of each group were selected randomly to weight (Table S6). Compared to the individuals in the control group with normal liver, most of individuals in the overfeeding group had bulge belly and randomly selected of them had fatty liver in general appearance after dissecting. Then, the liver samples of three individuals of the control group with normal liver and three individuals of the overfeeding group with fatty liver were collected randomly for histopathological observation and molecular analysis.

All fish were first totally immersed and deeply anesthetized with tricaine methanesulfonate (MS-222, 35–40 mg/L, Servivebio, Wuhan) until losing all rhythmic opercular movements for a minimum of 30 min, then sacrificed by rapidly cutting off the spinal cord adjacent to the head. Procedures were performed to minimize fish suffering as far as possible. All sections of this study were carried out in compliance with the ARRIVE guidelines [74], and a completed ARRIVE guidelines checklist was included (Checklist S1).

### Histological structure, lipid accumulation and morphological observation

Liver tissues were cut in sections and fixed in 4% paraformaldehyde (PFA) overnight at 4 °C, and subsequently embedded in paraffin. One part of sections was stained with hematoxylin & eosin (HE) (Beyotime, Suzhou) and performed as previously described [75, 76]. The Carl-Zeiss microscopy (Analytical & Testing Center, IHB, CAS) was used for histological structure observation and photomicrographs.

The other sections were used for detecting the lipid droplet morphology by ORO staining according to the previous method [77, 78]. The sections were totally dehydrated with 30% sucrose, and then embedded in optimum cutting temperature (OCT) compound. After being rapidly frozen and cut in Thermo CRYOSTAR NX50 microtome at 10 μm thickness, the sections were dyed with ORO (Thermo, USA) and counterstained with hematoxylin (Servivebio, Wuhan). Via the amount of ORO staining from 3 frames per biopsy, liver lipid accumulation was quantified using pixel numbers with Image J [79].

The specimens for morphological observation by electron microscopy were prepared as described previously [80, 81]. Liver tissues were fixed with 2.5% glutaraldehyde for 24 h at 4 °C, and in 1% osmium tetroxide (OsO4) for 2 h at 4 °C, and then gradiently dehydrated with ethanol. After that, sections were embedded in epoxy resin Epon812 for overnight and cut in Leica DMIRB ultrathin microtome at 60–80 nm thickness, stained with uranyl acetate and lead citrate, and observed with a HC-1 80.0 KV Hitachi TEM system (Analytical & Testing Center, IHB, CAS).

### RNA extraction, sequencing, assembly and functional annotation of liver transcriptomes

Three liver samples of two groups, normal liver and fatty liver, were listed as biological replicates (NL-1, NL-2, NL-3 and FL-1, FL-2, FL-3). Total RNA was extracted with RNeasy Mini Kit (Qiagen, Beijing) according to the manufacture’s protocols. Proper quality and quantity were checked with Nanodrop® ND-2000 spectrophotometer (LabTech, USA) and Technologies 2100 Bioanalyzer (Agilent Tech, USA) by measuring the 260/280 nm absorbance ratio. Fifty μg total RNA of each samples were used for cDNA library establishing and high-throughput sequencing via BGISEQ-500 platform in BGI Genomics Co., Ltd., Shenzhen, China. Clean reads were mapped to the gibel carp’ genome and genes using HISAT (v2.1.0) [82] and Bowtie2(v2.2.5) [83] (unpublished data). The expression levels of transcripts were calculated with RSEM [84]. Pearson’s correlation coefficient (r) and PCA were measured by using cor & princomp in R for the strength of the association between the two groups. DEG analysis was conducted by DEseq2 [85, 86] with q-value (adjusted *p*-value) ≤ 0.05. GO and KEGG pathway enrichment analysis were conducted with phyper in R. *p*-value was corrected with false discovery rate (FDR) cut-off of 0.01 and q-value ≤0.05 was used as the threshold to judge the significance of GO or KEGG enrichment. The raw sequences were deposited into the NCBI Sequence Read Archive (SRA) database (Accessions PRJNA675741).

### Latent pathways network construction and the significant pathways identification

Latent pathway identification analysis, LPIA, developed by Pham et al., is a method to identify the interactions of pathways associated with DEGs and biological processes [34, 64]. After preparing the three distinct but interrelated sources of biological pathways (such as “KEGG”, P), biological functions (such as “GO”, G) and gene transcriptional response in different conditions (such as “DEGs”, DE) (Table S4), 1) First, we constructed a P-G bipartite graph if P and G shares a non-empty intersection of DE, we calculated the weighted edge, denoted W_GP_, which stands for the intersection of P and G as follow

$$ {\mathrm{W}}_{GP}=\left(|\mathrm{G}\bigcap \mathrm{P}|\right)/\left(|\mathrm{G}\bigcup \mathrm{P}|\right)\times median\left\{{\mathrm{DE}}_x;x\in \mathrm{G}\bigcap \mathrm{P}\right\} $$

2) And then, converted the P-G bipartite graph to P-P pathway network if two pathways, P_i_ and P_j_ shares non-empty intersection of G, the weight between P_i_ and P_j,_ denoted A_i j_ is

$$ {A}_{ij}=\sum \limits_k^G{W}_{G_k{p}_i}\times {W}_{G_k{p}_j} $$

3) Finally, measured pathway importance via eigenvector centrality to determine the significance of a node in the above network after proper iterative operation. The weight scores > 0.1 were used to draw P-P pathway network with cytoscape 3.7.2 [87]. The whole process was repeated using the bootstrap method [88] and the adjusted *p*-values were calculated to account for multiple testing with methods described by Dudoit and van der Laan [89].

### Quantitative reverse transcription PCR (qPCR)

The first-strand cDNA was synthesized from total RNA following the protocol of Thermo Scientific™ RevertAid First Strand cDNA Synthesis Kit (Thermo Fisher Scientific, USA) in a 20 μl reaction volume. The expression level was analyzed on a CFX96™ Real-Time PCR System (Bio-Rad, USA) using an iTaqTM Universal SYBR®Green Supermix (Bio-Rad, USA). Gene-specific primers (Table S8) were designed with Primer premier 5.0 [90]. The reaction system, protocol, and endogenous control selection were conducted as previously described [91–93]. All liver samples were analyzed with three biological replicates and the relative expression levels of target genes were normalized to β-actin and calculated by the 2^-∆∆CT^ method. *p*-value < 0.05 was considered statistically significant.

## Supplementary Information

**Additional file 1: Figure S1** PCA analysis and relevant heat map of six liver samples. **a** PCA plot of DEGs among liver samples. Yellow circle: samples in PC1 belong to fatty liver. Blue circle: samples in PC2 belong to normal liver. **b** Relevant heat map of six liver samples. NL: normal liver, FL: fatty liver.

**Additional file 2: Table S1** DEGs (FDR ≤ 0.001) in normal liver and fatty liver. Lists of DEGs include Gene ID, Length, FPKM, Log_2_ fold change, Q-value, *P-* value, and Annotation.

**Additional file 3: Table S2** GO terms with all up/downregulated DEGs enrichment analysis. GO terms with all upregulated DEGs (GO terms with all up-DEGs), GO enrichment analysis with all upregulated DEGs (enriched GO-up), GO terms with all downregulated DEGs (GO terms with all down-DEGs) and GO enrichment analysis with all downregulated DEGs (enriched GO-down). NL: normal liver, FL: fatty liver.

**Additional file 4: Table S3** KEGG pathway enrichment analysis. Pathway ID, Pathway Name, KEGG function classification, number of Candidate and total DEGs/genes, Rich Ratio, *P*-value, Q-value are shown. NL: normal liver, FL: fatty liver.

**Additional file 5: Figure S2** The left three latent pathways. **a** Porphyrin and chlorophyll metabolism pathway (23 DEGs, ko00860, https://www.genome.jp/dbget-bin/www_bget?map00860). **b** HIF-1 signaling pathway (49 DEGs, ko04066, https://www.kegg.jp/kegg-bin/show_pathway?map05211). **c** Mineral absorption pathway (17 DEGs, ko04978, https://www.genome.jp/dbget-bin/www_bget?map04978) in comparative transcriptomic analysis. Up and downregulated DEGs are shown in red and green, respectively.

**Additional file 6: Table S4** Input and output files in LPIA. Biological pathways (FL_NL_P), biological functions (FL_NL_G) and gene transcriptional response in different conditions (FL_NL_DE) are used in LPIA and the output file (Output_LPIA_FL_NL). NL: normal liver, FL: fatty liver.

**Additional file 7: Table S5** Pathway-pathway network. The pathways include 43 enriched pathways in KEGG enrichment analysis and four significant pathways in LPIA method.

**Additional file 8: Table S6** Growth performance of gibel carp in the Luo-Fu Lotus Farm. Lists of parameters include: initial body weight (IBW), final body weight (FBW) and specific growth rate (SGR).

**Additional file 9: Table S7** Aquaculture conditions in the Luo-Fu Lotus Farm. Lists of conditions include Water temperature (WT), dissolved oxygen (DO), total nitrogen (TN), ammonia (NH_4_-N), nitrate (NO_3_-N), nitrite (NO_2_-N), and total phosphorus (TP).

**Additional file 10: Checklist S1** Completed ARRIVE guidelines checklist**.** The checklist includes the ARRIVE Essential 10 and the Recommended Set.

**Additional file 11: Table S8** Primers used in this study.

## Data Availability

The raw sequences supporting the conclusions of this article were deposited into the NCBI Sequence Read Archive (SRA) database under the accession number PRJNA675741(NL: SRR13023909, SRR13023907 and SRR13023906; FL: SRR13023911, SRR13023910 and SRR13023908).

## Abbreviations

AMPK: AMP-activated protein kinase
acsl4: Acyl-CoA synthetase long-chain family member 4
BP: Biological processes
CC: Cellular component
DEG: Differential gene expression
FBW: The final body weight
FDR: False discovery rate
GO: Gene Ontology
gcl: Glutamate-cysteine ligase
gpx4: Glutathione peroxidase 4
HE: Hematoxylin & eosin
NAFLD: Nonalcoholic fatty liver disease
JNK: C-jun Nterminal kinase
LPIA: Latent pathway identification analysis
KEGG: Kyoto Encyclopedia of Genes and Genomes
MF: Molecular functions
Myd88: Myeloid differentiation primary-response protein 88
NF-κB: Nuclear factor κB
Nrf2: Nuclear erythroid 2-related factor 2
OCT: Optimum cutting temperature
ORO: Oil red o
PCA: Principal component analysis
PFA: Paraformaldehyde
qPCR: Quantitative reverse transcription PCR
ROS: Reactive oxygen species
SGR: Specific growth rate
TFEB: Transcription factor EB
tfr1: Transferrin receptor 1
TLRs: Toll-like receptors
Torc1: Target-of-rapamycin complex 1
