# Supplementary figures and images for: Comparative transcriptomic analysis reveals an association of gibel carp fatty liver with ferroptosis pathway
Source: BMC Genomics. 2021 May 5;22:328. doi: 10.1186/s12864-021-07621-2 (PMC8101161; doi:10.1186/s12864-021-07621-2)

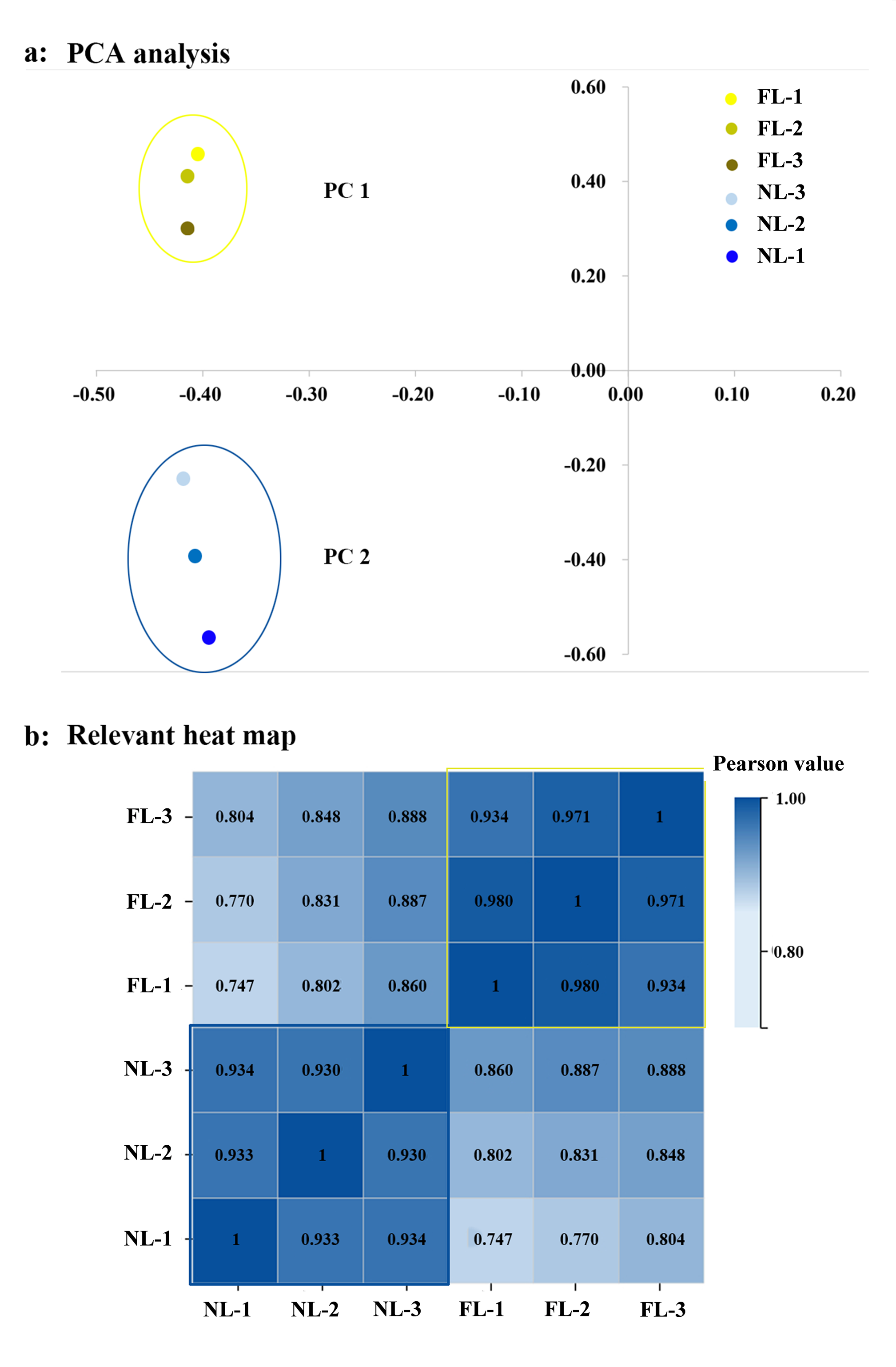

Supplement: Supplementary file 1 — Additional file 1: Figure S1 PCA analysis and relevant heat map of six liver samples. a PCA plot of DEGs among liver samples. Yellow circle: samples in PC1 belong to fatty liver. Blue circle: samples in PC2 belong to normal liver. b Relevant heat map of six liver samples. NL: normal liver, FL: fatty liver. [file 12864_2021_7621_MOESM1_ESM.tif]

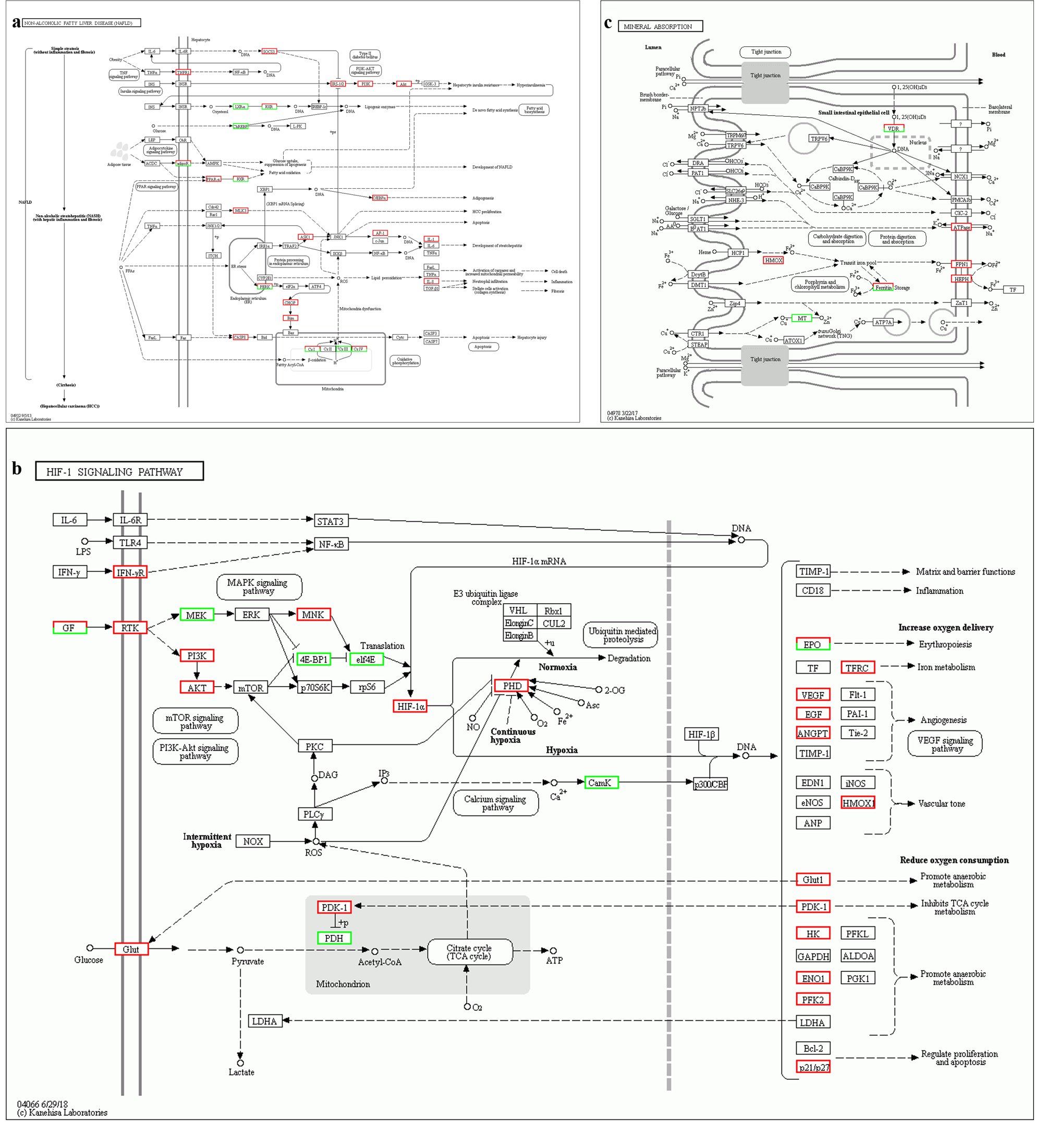

Supplement: Supplementary file 5 — Additional file 5: Figure S2 The left three latent pathways. a Porphyrin and chlorophyll metabolism pathway (23 DEGs, ko00860, https://www.genome.jp/dbget-bin/www_bget?map00860). b HIF-1 signaling pathway (49 DEGs, ko04066, https://www.kegg.jp/kegg-bin/show_pathway?map05211). c Mineral absorption pathway (17 DEGs, ko04978, https://www.genome.jp/dbget-bin/www_bget?map04978) in comparative transcriptomic analysis. Up and downregulated DEGs are shown in red and green, respectively. [file 12864_2021_7621_MOESM5_ESM.tif]
